# Utilizing the virus-induced blocking of apoptosis in an easy baculovirus titration method

**DOI:** 10.1038/srep15487

**Published:** 2015-10-22

**Authors:** Athanasios Niarchos, George Lagoumintzis, Konstantinos Poulas

**Affiliations:** 1 Department of Pharmacy, University of Patras, GR 26500, Patras, Greece

## Abstract

Baculovirus-mediated protein expression is a robust experimental technique for producing recombinant higher-eukaryotic proteins because it combines high yields with considerable post-translational modification capabilities. In this expression system, the determination of the titer of recombinant baculovirus stocks is important to achieve the correct multiplicity of infection for effective amplification of the virus and high expression of the target protein. To overcome the drawbacks of existing titration methods (*e.g.,* plaque assay, real-time PCR), we present a simple and reliable assay that uses the ability of baculoviruses to block apoptosis in their host cells to accurately titrate virus samples. Briefly, after incubation with serial dilutions of baculovirus samples, Sf9 cells were UV irradiated and, after apoptosis induction, they were viewed via microscopy; the presence of cluster(s) of infected cells as islets indicated blocked apoptosis. Subsequently, baculovirus titers were calculated through the determination of the 50% endpoint dilution. The method is simple, inexpensive, and does not require unique laboratory equipment, consumables or expertise; moreover, it is versatile enough to be adapted for the titration of every virus species that can block apoptosis in any culturable host cells which undergo apoptosis under specific conditions.

Baculovirus-based protein expression systems combine high yields with considerable post-translational modification capabilities. Thus, baculovirus-mediated protein expression is a robust experimental technique to produce recombinant proteins of higher eukaryotic origin, especially when heterologous expression in bacteria fails^1^. In the baculovirus-mediated expression system, knowledge of the titer of recombinant baculovirus stocks is important to achieve the correct multiplicity of infection, which is the most critical factor for effective amplification of the virus and high expression of the target protein^2^. Therefore, many baculovirus titration methods have been developed. Plaque assay^3^, end point dilution assay^4^, antibody-based assays^5^,^6^, flow cytometry assays^7^,^8^, quantitative real-time PCR^9^, and assays utilizing dye-substrates for metabolic enzymes (*e.g.*, AlamarBlue^®^)^10^ are among the other methods that have been developed and are extensively used. Although these assays are very useful, each of them also has some drawbacks. For example, some of them are labor-intensive and time-consuming (*e.g.*, plaque assay), or require unique skills and expertise (*e.g.*, for recognition of virus-positive wells in the end point dilution assay), whereas other titration assays use expensive consumables, expensive equipment, or both (*e.g.*, quantitative real-time PCR and flow cytometry based methods).

In the current study, a new and versatile method is proposed that does not have the disadvantages of the aforementioned methods for virus titration. The method combines simplicity, reliability, and commonly used, low cost consumables, and it is very easy to apply without the need for expensive equipment. The proposed method is based on the ability of baculoviruses to block apoptosis in their host cells^11^,^12^,^13^; thus, we call it Apoptosis Blocking Assay (ABA). Apoptosis is a process of programmed cell death that is common among multicellular organisms, which ultimately turns apoptotic cells into vesicles^14^. In this study, ABA was developed and applied to the titration of the baculovirus species *Autographa californica* multiple nucleopolyhedrovirus (*Ac*MNPV), which is widely used for gene expression in insect cells^2^. Cultured attached Sf9 insect cells in 96-well tissue culture plates were infected with serial dilutions of the baculovirus stock, and after 48 hours of incubation they were UV-irradiated to induce apoptosis^15^,^16^. After another 16-hour incubation, the plates were examined microscopically for islets of intact, infected cells in which apoptosis had been blocked (virus-positive wells) among apoptotic vesicles derived from the non-infected cells. Thereafter, virus titers were calculated using the Reed and Muench method. Finally, baculovirus titers determined using ABA were compared with the respective titers determined by end point dilution assay (EPDA).

## Results

### ABA for baculovirus

**Figure 1:**
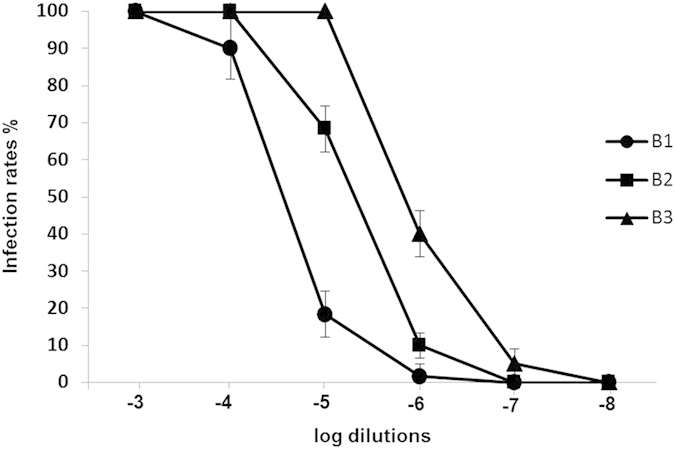
Percentage infection rates scored by increasing dilutions of baculovirus batches B1, B2 and B3 using ABA. The % average infection rate values are presented along with their standard deviation values (*n *= 5) versus the log values of increasing B1, B2 and B3 dilutions.

**Figure 2:**
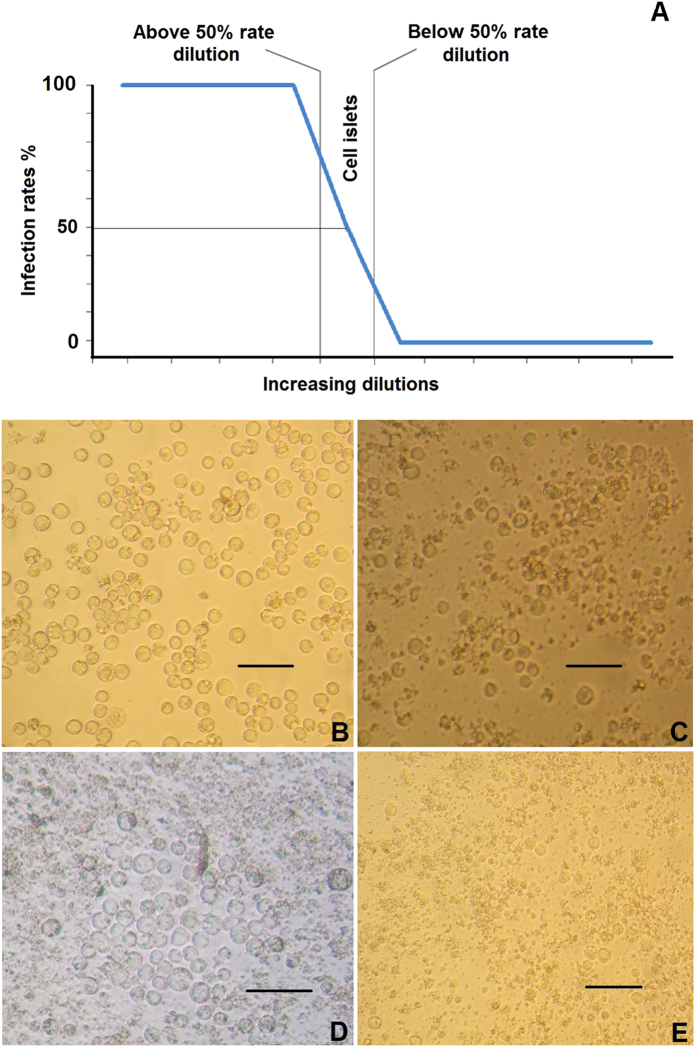
Infection rates and cell appearance in ABA, as a result of inoculation with lower and higher virus dilutions. (**A**) Curve of % infection rates vs. dilution of viral inoculum. Cell appearance after UV irradiation and 16 hours incubation after inoculation with: (**B**) low virus dilutions, (**C**) higher virus dilutions, (**D**) dilutions next above and next below the 50% infection rates (islet formations of the remaining cells), and (**E**) very high dilutions that do not contain virions. Scale bars = 50 μm.

**Figure 3:**
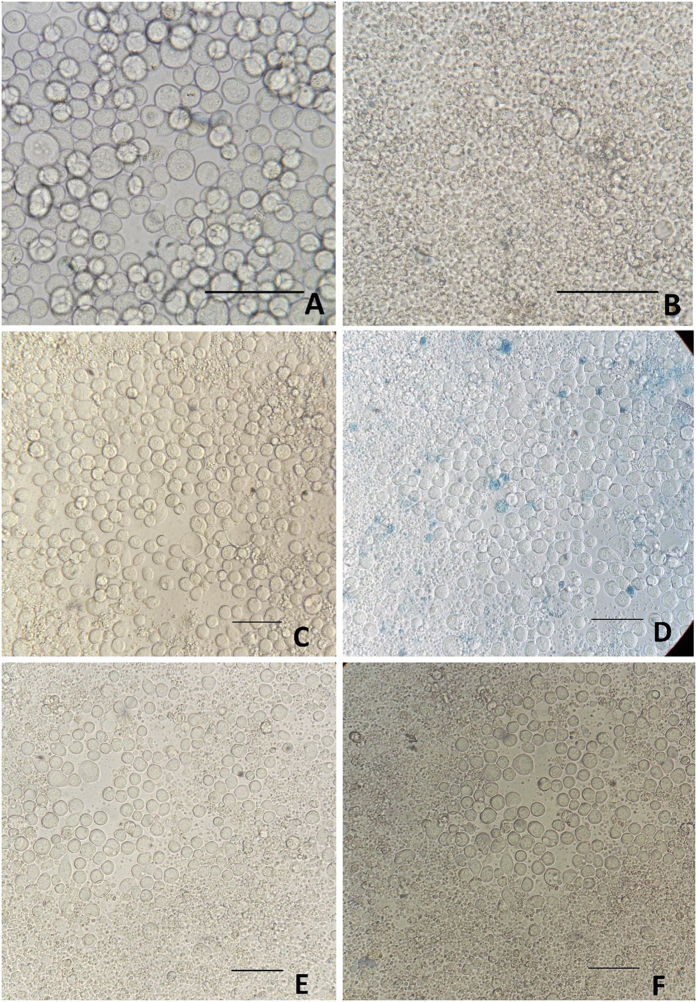
Trypan blue staining and stability evaluation of cell islets. (**A**) Untreated, uninfected Sf9 cells, (**B**) Sf9 cells uninfected, after UV irradiation and the following apoptosis, (**C**) cell islet untreated and (**D**) the same cell islet after Trypan blue staining. (**E**) Cell islet after UV irradiation and apoptosis and (**F**) the same islet 5 days later. Scale bars = 50 μm.

To determine the titers of baculovirus batches B1, B2 and B3 using ABA, the virus-positive wells were presented as % infection rates (Fig. 1). Figure 1 shows that the infection rates for all dilutions were decreased and followed the same pattern, irrespective of baculovirus batch. This pattern is presented graphically in Fig. 2A. Wells inoculated with low viral dilutions presented infection rates of 100%; after UV irradiation and 16 hours of incubation, cells appeared microscopically intact (as in Fig. 2B) because they were all infected and their apoptosis mechanism was blocked. In wells inoculated with higher viral dilutions, the proportion of intact infected cells reduced rapidly and they appeared as in Fig. 2C. In wells inoculated with even higher viral dilutions (the infection rates next above and next below 50%) (Fig. 2A), intact infected cells with blocked apoptosis mechanisms appeared as islets (Fig. 2D). These islet-like formations (50–100 cells) most likely resulted from the release of virions from single infected cells, which subsequently infected neighboring cells. In wells inoculated with even higher viral dilutions that did not contain any virion, the infection rates decreased to zero (Fig. 2A), and only very few scattered intact cells were observed after apoptosis completion (Fig. 2E). Non baculovirus inoculated cells (Fig. 3A) when UV-irradiated gave exactly the same picture (Fig. 3B) after apoptosis completion. The remained cell islets appeared to be negative to Trypan blue staining (Fig. 3D). After the initial observation by microscopy of non-Trypan blue stained wells (Fig. 3E), the same wells were imaged again after five additional days of incubation, and absolutely no changes were observed (Fig. 3F).

### Evaluation of ABA for baculovirus titration

**Figure 4:**
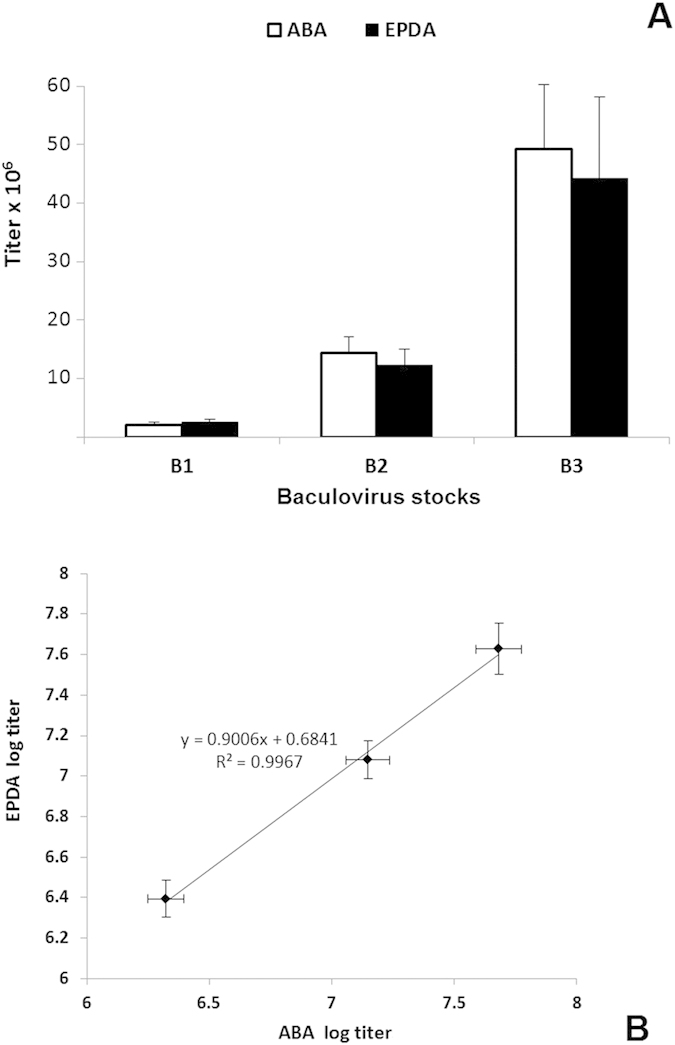
Evaluation of ABA for baculovirus. (**A**) The three baculovirus stocks were tittered (five times each) using ABA (white columns) and EPDA (black columns), and the average titer values are presented along with their standard deviation values. (**B**) Correlation between the log titers of each of the three baculovirus stocks, determined using ABA and EPDA. The line in the diagram represents a linear regression fit to the data (mean ± SD, *n *= 5). The correlation coefficient (R^2^) was calculated to be 0.9967.

To evaluate ABA as a titration method, the three baculovirus stock titers were determined using ABA and EPDA. Five separate experiments were performed for B1, B2 and B3 using each of the two techniques, and the mean and the standard deviation of the titers from the two techniques were calculated and plotted in Fig. 4A. The diagram shows that ABA and EPDA gave similar titers for B1, B2 and B3 (±14% on average), whereas the average variance of this particular ABA protocol was calculated to be ±20%. The correlation (R^2^) between the log titer values determined using ABA and EPDA was 0.9993 (Fig. 4B).

## Discussion

Baculovirus-based protein expression systems are often used to produce proteins originating from higher eukaryotic organisms. The determination of virus stock titers in baculovirus protein expression systems is essential for successful virus amplification and high protein expression^2^. To date, many different methods have been developed for baculovirus titration, but each one of these methods has its drawbacks. In this study, a new, simple and reliable assay is proposed and applied to baculovirus titration, utilizing virus-induced blocking of apoptosis. To induce apoptosis in the non-infected cells, UV light from a commercially available UV lamp for DNA visualization was used, although any other convenient protocol ensuring the maximum degree of apoptosis of the non-infected cells in every single well of a 96-well plate can be used (*e.g.,* chemical induction of apoptosis)^17^.

A critical requirement for baculovirus ABA, as well as the accurate preparation of dilutions, is the use of healthy, early passage and fast growing (generation/doubling time ~36 hours) Sf9 cells that allow efficient infection and amplification of baculovirus^18^. If the cells are not healthy or if they are old, ABA will not perform properly. For Sf9 cells with slightly slower growth rates, *e.g.*, Sf9 cells cultured without FBS, a 3-day infection could be more efficient than a 2-day infection.

In the current study, the proposed ABA combines several advantageous characteristics that, in our opinion, render it an attractive alternative to other established methods. This technique is as simple as EPDA^19^ to set up, but it is easier to define the virus-positive wells and much faster (over the weekend in 60–64 h vs. 5–7 days for EPDA). In contrast to the classic plaque assay^3^ and other techniques that use expensive consumables, such as immunological^5^,^6^ and quantitative real-time PCR techniques^20^,^21^, ABA does not require any supplies other than those that are necessary for cell culture and apoptosis induction. As well as the economic advantages over the routinely used techniques that rely on expensive equipment and consumables, ABA also has the advantage of measuring only the useful infective virions.

Other strengths of ABA are that it can be performed without specific expertise, and it does not require a standard virus batch of known titer. In the present study, the protocol used for baculovirus ABA was found to have an average consistency of ±20%, which is the average level of consistency for the majority of the established methods^22^. In our study, the baculovirus titers determined by ABA were crosschecked against those determined by EPDA and the titers determined by both assays were identical with an average deviation of ±14.4%. Furthermore, the results from the two methods were found to be statistically correlated, with a correlation coefficient value of 0.9967 between the log titer values determined by the two techniques. Finally, it is important to emphasize that ABA is inherently versatile; it can be adapted to titrate every virus that blocks apoptosis in any culturable cells^11^,^13^ which undergo apoptosis under specific conditions^17^. Considering all of the above factors, we believe that ABA could be a valuable general-use titration method for baculovirus and other viruses.

## Methods

### Insect cell cultures

Sf9 insect cells (Invitrogen) were suspension-cultured at 27 °C, starting from a density of 5 × 10^5^ cells/ml, in SF900 III medium (Invitrogen) supplemented with 5% heat-inactivated FBS (Gibco) , under constant agitation on a rocker at 15 rpm, up to a density of 5 × 10^6^ cells/ml. Suspension cultures were diluted with SF900 III/5% FBS up to 10^5^ cells/well and, subsequently, 100 μl of the diluted suspension cultures was dispensed into each well of a 96-well tissue culture plate (Greiner Bio-One) and incubated for 1 h at 27 °C in a humidified incubator, enabling the cells to attach prior to subsequent experiments.

### Baculovirus batches

Baculovirus batches 1, 2 and 3 (B1, B2 and B3, respectively) were produced after transfection of Sf9 insect cells with the baculovirus genome “bacmid” (bMON14272) from the Bac-to-Bac^®^ expression system (Invitrogen) according to the manufacturer’s protocol.

### ABA for baculovirus

Six 1:10 serial dilutions of B1, B2 and B3, starting from 10^−3^, were prepared, and 10 μl from each dilution was added gently, to avoid disturbing the 10^4^ attached cells, into each of the 12 wells along each row (1 row for each dilution). The plates were incubated for 2 days and then UV-irradiated for 2 min directly on the top of a 6-Watt 312 nm UV lamp (Kisker) in a dark room, enclosed in aluminum foil and incubated for 16 hours to allow apoptosis to be completed. Then, the plates were examined microscopically for virus-positive wells containing islets of intact infected cells in which apoptosis had been blocked. Two days of infection was the minimum duration to accurately discriminate the virus-positive from the virus-negative wells. For evaluation of ABA, 5 separate experiments were performed for each baculovirus batch.

### Trypan Blue staining

One hundred and ten μl of 0,05% Trypan Blue solution in PBS were added in wells containing cell islets, after UV irradiation and apoptosis completion. After 5 minute incubation, cells were examined microscopically.

### End point dilution assay (EPDA)

The same baculovirus dilutions were used for EPDA as for ABA, and the same volume was added (*i.e.*, 10 μl) per well, in the same number of wells (*i.e.*, 12 for each dilution) and for the same number of experiments (*i.e.*, 5). Ninety-six-well plates were incubated for 4 days and examined microscopically. After the cells were detached by pipetting, 10 μl of cell suspension from each well was subcultured in a new well containing 100 μl of SF900 III/5% FBS. After another 4-day incubation, plates were examined microscopically for cell proliferation and, after cell detachment by pipetting, 10 μl from each well was transferred to a new well containing 100 μl of SF900 III/5% FBS, incubated for 2 hours and examined for cell attachment and morphology. The cells’ inability to proliferate and attach as well as the presence of morphological lesions/distortions implies baculovirus infection; thus, specific wells were deemed to be baculovirus-positive. Wells that displayed high levels of cell proliferation and cell attachment and normal cell morphology were deemed to be baculovirus-negative. Subsequently, the batch titers were calculated as for ABA. Subculturing and attachment tests were performed to render the distinction between virus-positive and virus-negative wells objective and reliable.

### Calculation of B1, B2 and B3 titers

For the calculation of baculovirus batch titers in ABA and EPDA, the % proportion of virus-positive wells among the number of inoculated wells (*i.e.*, 12) was determined to be the infection rate of every batch dilution. Subsequently, the titer for every virus batch was calculated according to the Reed and Muench method. From the dilutions that scored infection rates next above and next below 50%, the proportion distance (PD) was calculated using the formula
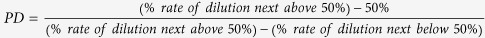


PD was used to calculate the 50% endpoint dilution (endpoint) from the equation


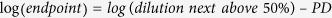
. The batch titers were calculated using the formula 
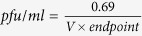
 , where pfu: plaque forming units, V: volume of added viral dilution.

### Statistical analysis

The mean and standard deviations of the experimentally determined values are presented graphically. For each baculovirus batch, five replicate ABA and five replicate EPDA experiments were carried out, and the average and the % standard deviations of the obtained titers were calculated. Each baculovirus batch titer was determined as the average of titers determined from the five replicate experiments (either ABA or EPDA), whereas the consistency of ABA was determined as the average of the % standard deviations of the three batch titers. All calculations were conducted using Microsoft Excel.

## Additional Information

**How to cite this article**: Niarchos, A. *et al.* Utilizing the virus-induced blocking of apoptosis in an easy baculovirus titration method. *Sci. Rep.* **5**, 15487; doi: 10.1038/srep15487 (2015).
